# The effects of personality traits on learning engagement among college students: the mediating role of emotion regulation

**DOI:** 10.3389/fpsyg.2024.1476437

**Published:** 2025-01-10

**Authors:** Tongtong Dang, Wenxiu Du, Menghui Niu, Zhenguo Xu

**Affiliations:** ^1^Faculty of Education, Qufu Normal University, Qufu, China; ^2^School of Communication, Qufu Normal University, Rizhao, China

**Keywords:** personality traits, Big Five Personality Traits, learning engagement, emotion regulation, mediating role

## Abstract

**Introduction:**

As we all know, learning engagement is a key indicator for measuring the quality of students’ learning outcome and assessing their learning effectiveness. However, the relationship among personality traits, emotion regulation, and learning engagement has not been thoroughly studied.

**Methods:**

This study aims to investigate the relationship among personality traits, emotion regulation and learning engagement. A questionnaire survey was conducted on some college students in Shandong Province, China.

**Results:**

The results show that: (1) there are no significant differences in Big Five Personality Traits, learning engagement, and emotion regulation by gender, grade level, and subject category to which the major belongs. (2) The personality traits of college students can directly affect learning engagement. (3) Emotion regulation partially mediates the relationship between personality traits and learning engagement.

**Discussion:**

The study reveals the importance of personality traits and emotion regulation on students’ learning. It has a significant meaning in enhancing students’ learning engagement level.

## 1 Introduction

Higher education is an important link in building a strong educational country, hence, it becomes one of the key concerns of researchers at home and abroad. Higher education is committed to cultivating high-level specialists who can master professional knowledge and skills. Learning engagement is an important educational outcome for students in the 21st century (Fullan et al., 2018). The essence of higher education is learning engagement and student development (Hu, 2005). Research has shown that learning engagement is closely related to college students’ academic achievement and growth experience, what’s more, it is a predicting indicator of higher education quality (Axelson and Flick, 2010; Liu, 2015; Wu et al., 2023). Personality traits refer to the psychological structure as well as behavioral responses made by individuals under different kinds of stimuli (Zhao et al., 2019). Compared with intelligence level, personality traits are more predictive of students’ academic performance (Goff and Ackerman, 1992). Understanding the personality traits of college students is an important prerequisite for improving college students’ learning achievement and optimizing their growth experience. Emotions are reactions to situations, actions, or events. During the learning process, emotions inevitably fluctuate (Ahn and Harley, 2020). Emotional fluctuations are responses to the learning process, affecting students’ learning experience and learning outcomes (Fuente et al., 2024; Chen-Bouck et al., 2024). Although there are previous studies that have examined the relationship between personality traits and learning engagement (Mahama et al., 2022; Zhao and Ji, 2024; Morfaki and Skotis, 2022), as well as studies studying on emotion regulation for learning engagement (Pan, 2023; Huang et al., 2023; Zhang et al., 2024) and studies on the role of emotion regulation as a mediator (Zhang et al., 2024; Li et al., 2024; Qiao et al., 2024), there are relatively few studies on the relationships among personality traits, emotion regulation, and learning engagement.

This study aims to design reliable scales to collect data, use authoritative tools to analyze the data, and construct a model of the influencing factors of college students’ personality traits, emotion regulation, and learning engagement through scientific methods in order to deeply explore and reveal the intrinsic correlations among these three items. With the help of this study, the study benefits can help learners learn more easily and happily, and have them actively participate in various learning activities, so as to further promote the overall improvement of the quality of teaching and learning in higher education.

## 2 Theoretical review

### 2.1 Conceptualization and dimensions of learning engagement

The term learning engagement can be traced as far back as the concept of Time on Task proposed by Ralph Tyler, who argued that success is directly proportional to the time invested (Madaus and Stufflebeam, 1989). According to Liu et al. (2017), learning engagement refers to the positive mindset adopted by an individual during the learning process. According to Astin (1984), learning engagement is the sum of physical and mental energy that students put into their coursework activities. Towards this issue, scholars also have their own views on the dimensional division of learning engagement, hence, expressing their respective viewpoints. Finn and Zimmer (2012) classified learning engagement into four levels, with Level I learning engagement characterized by students simply complying with the basic requirements posed by school, e.g., attending classes. Level II learning engagement is characterized by enthusiasm and initiative input for academic tasks. Level III learning engagement occurs outside the formal classroom and represents student engagement in extracurricular activities. Level IV learning engagement includes goal setting, decision making, and assuming leadership roles. According to Schaufeli et al. (2002), learning engagement is a positive and sustained affective state exhibited by students during the learning process. It is characterized by vigor, dedication, and focus. Kuh et al. (2007) believes that learning engagement has two dimensions. One is the students’ own engagement in activities that promote their academic success. The second is that students engage in activities and contribute to their own improvement through the help of their belonging schools. However, Fredricks and McColskey (2012) argued that learning engagement consists of three dimensions: cognitive engagement, behavioral engagement, and affective engagement. Cognitive engagement mainly includes problem-solving strategies, metacognitive strategies, and so on. Behavioral engagement mainly includes persistence, effort, and attention. Affective engagement mainly includes interest, pride, and so on. After that, Fredricks et al. (2016) gave consideration to the effect of social interaction among students on learning engagement. He proposed that learning engagement includes cognitive engagement, behavioral engagement, affective engagement, and social engagement. Other researchers such as Christenson et al. (2012) pointed out that learning engagement consists of four dimensions: behavioral engagement, cognitive engagement, affective engagement, and initiative engagement. The four aspects with each other and are related to each other. Therefore, based on all these viewpoints, this study argues that learning engagement refers to the active, fulfilling, and sustained state that students exhibit in the learning process. It not only includes the time, energy, and resources that students put into their academics, but also involves students’ investment in multiple dimensions such as affective, cognitive, and behavioral. This study adopts a three-dimensional framework of behavioral engagement, affective engagement, and cognitive engagement to study learning engagement.

### 2.2 Conceptualization, dimensionality and impact of personality traits on learning engagement

Personality traits are conceptualized as neurophysiological systems that induce adaptive expressive behaviors in response to stimuli (Allport, 1961). Personality traits are the active behavioral responses that people make to their environment. Scholars do not have a recognized universal standard for defining personality traits. Therefore, they began to revert the directions of their researches from the definition of personality traits to the classification of personality traits. Allport (1961) believed that traits are the root of personality. It is the individual’s own traits and not the environment that determines his behavioral activities. He categorized personality traits into primary, central and secondary traits. Eysenck and Eysenck (2013) utilized the means of trait clusters to categorize personality traits into extraversion, neuroticism, and psychoticism. Cattell (1943) used factor analysis to categorize personality traits into two dimensions: surface traits and root traits. McCrae and Costa (2004) suggested that personality traits consist of five major dimensions, which are extraversion, neuroticism, openness, conscientiousness, and agreeableness. Individuals with high extroversion are good communicators and are prone to more easily acquire pleasure and satisfaction in socialization. Neuroticism reflects emotional stability; individuals high in neuroticism are emotionally highly reactive and exhibit impulsive temper. Openness mainly refers to an individual’s exploration of unfamiliar situations. Individuals with high openness are characterized by high creativity and strong divergent thinking. Conscientiousness refers to the individual’s self-control. Individuals with high conscientiousness are well organized and persistent in their work. Agreeableness refers to an individual’s attitude toward others. Individuals with high agreeableness show empathy and are more willing to help others. Above all, these are referred to as the Big Five Personality Traits. The Big Five Personality Traits are highly representative, therefore, this study uses the Big Five Personality Traits to categorize personality traits. Scholars have also studied the relationship between personality traits and learning engagement. Su (2023) collected questionnaires from senior nursing students and found that senior nursing students’ learning engagement was significantly negatively correlated with neuroticism, and significantly positively correlated with the other four personality traits. Jia (2023) investigated middle school students through the Proactive Personality Scale and Learning Engagement Scale and found that proactive personality directly predicted very good learning engagement and also positively greatly predicted learning engagement through academic resilience. Fan (2021) explored the influencing factors affecting college students’ learning engagement through Nvivo 11 software and found that personality traits are key factors affecting college students’ learning engagement. Jiang et al. (2018) found that perseverance personality positively predicted learning engagement as well as academic achievement. They had distributed questionnaires to primary and secondary school students, discovering that learning engagement fully mediated the role of perseverance personality and academic achievement. Cheng et al. (2022) concluded that the Big Five Personality Traits have a significant impact on students’ motivation to learn, and their attributional styles partially mediate the effect. Kara et al. (2024) used the Student Engagement Scale in Online Learning Environments to survey 437 college students and found that the personality trait was a significant predictor of student engagement in online learning environments. Wu and Yu (2023) collected data from 1,004 university students and used structural equation modelling to explore how students with different personality traits engage in e-learning. Qureshi et al. (2016) explored the predictive role of personality in multidimensional engagement models by surveying students and employees. In addition, the studies of several experts such as Mahama et al. (2022), Ongore (2014), Qureshi et al. (2016), and Bao et al. (2022) have shown that learning engagement is affected by personality traits. Therefore, this study used personality traits as variables, utilized Big Five Personality Traits Scale and Learning Engagement Scale to investigate the relationship between the influence of personality traits and learning engagement among college students.

### 2.3 Conceptualizations, dimensions and effects of emotion regulation on learning engagement

In our daily lives, we produce a wide range of emotions, and emotions are an individual’s response to the environment (Shi, 2023). Emotion regulation is a kind of mental activity processing engineering, which can influence what emotions an individual produces and the duration of generated emotions (Thompson and Goodman, 2010). Emotions are regulatable and individuals can regulate their emotions through conscious or unconscious behaviors (Li, 2019). Scholars have found that emotion regulation has an impact on learning engagement. Zhang and Wang (2023) found that academic emotions play a mediating role in the relationship between teacher support and foreign language learning engagement by establishing a multiple mediation model, which can significantly and indirectly predict foreign language learning engagement through emotions such as pleasure and anxiety. Liu et al. (2023) found that students’ positive emotions in a blended learning environment had a positive effect on deep cognitive engagement by carrying out through a questionnaire survey. Xu et al. (2023) found that the correlation among self-efficacy, emotional engagement and learning engagement was significant by interviewing 563 college students through a questionnaire survey, which further proved that emotion regulation partially mediated the relationship between self-efficacy and learning engagement. Zhang et al. (2024) conducted an in-depth survey of 1,506 high school English as a Foreign Language (EFL) learners using a questionnaire, and the results of the study showed that emotional inhibition mediated the relationship between perceived peer support and EFL behavioral engagement, i.e., when learners perceive a decrease in support from their peers, they may respond by inhibiting their positive emotions, which in turn affecting their behavioral engagement in EFL. Another quasi-experimental study conducted by Arguedas et al. (2016) further confirmed the positive impact of emotion regulation on learning outcomes. The study targeted a group of high school students and guided them through specific teaching strategies to become aware of their emotional states and learn how to regulate and manage these emotions effectively. The results of the study showed that when students were able to accurately identify and regulate their emotions, their motivation, engagement, and self-regulation were significantly enhanced, thus contributing to improved academic performance. Xiao et al. (2024) found that cognitive reappraisal was positively correlated with learning engagement through the use of a questionnaire targeting 1,200 high school students and that cognitive reappraisal mediated the relationship between Internet addiction and learning engagement. All these studies have emphasized the critical role of emotion regulation strategies in academic engagement. What’s more, some studies have shown that personality traits affect an individual’s emotion regulation (Qu and Wang, 2024). Therefore, this study adopted emotion regulation as a mediating variable to investigate the effect of emotion regulation on learning engagement and the mediating role of emotion regulation in personality traits and learning engagement.

### 2.4 Present study and hypotheses

**Figure 1:**
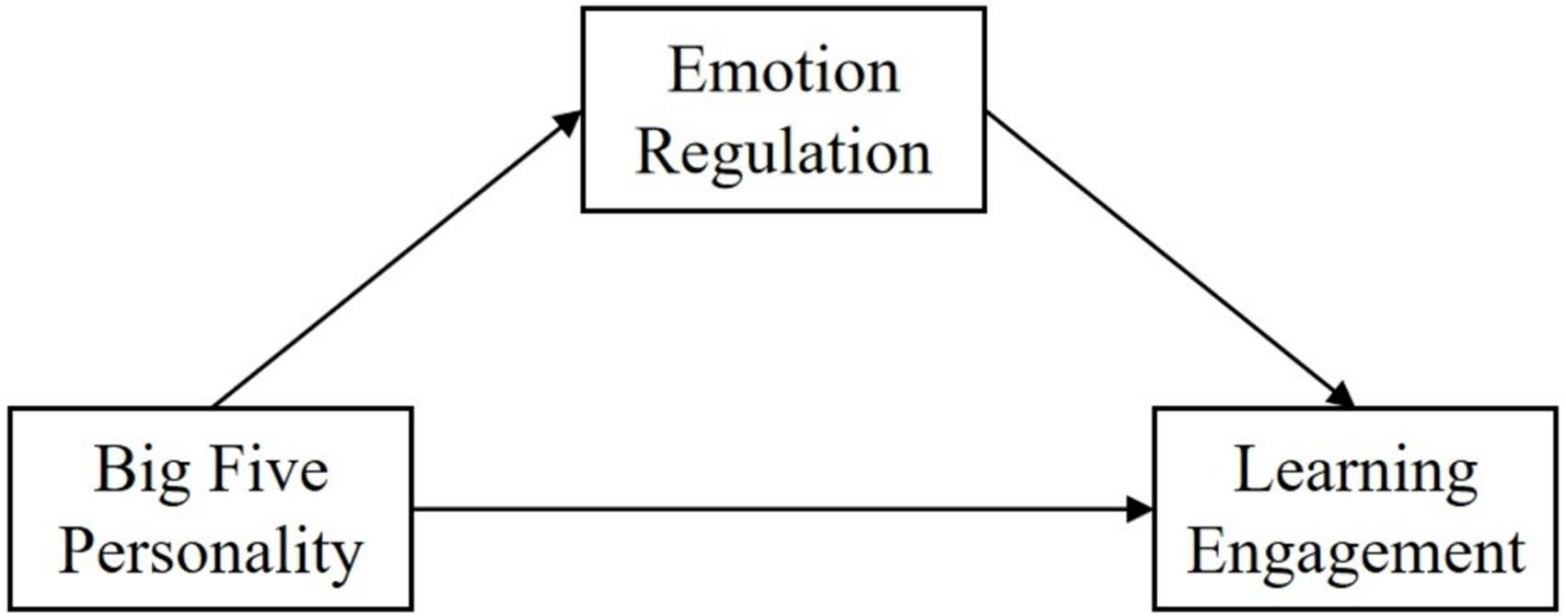
Hypothetical model of the relationship between personality traits and learning engagement.

Previous research has demonstrated that personality traits, and emotion regulation all have an impact on learning engagement. However, few researchers have put the three together to study their influence mechanisms. Studying their influence mechanisms has a significant importance for improving students’ emotions and enhancing learning engagement. Therefore, this study takes college students as the research object to investigate the relationship between personality traits and learning engagement, and expound the mediating role of emotion regulation. Based on the results of existing studies, the following hypotheses and conceptual model were proposed in this study (Figure 1).

Hypothesis 1 (*H1*): Personality traits will directly affect students’ learning engagement.

Hypothesis 2 (*H2*): Emotion regulation will mediate the relationship between personality traits and learning engagement.

## 3 Materials and methods

### 3.1 Participants and procedure

In this study, some universities in Shandong Province, China, were selected as participating units through convenience sampling method, and college students were treated as the research objects. The wide range of subjects’ specialties makes the collected data more real and reliable. The backend system shows that a total of 251 questionnaires were distributed, while 243 questionnaires were recovered, with a recovery rate of 96.8%, and 235 questionnaires were valid, with an effective rate of 96.7%. The age of the participants ranged from 19 to 23 years old. The average was 21 years old. The oldest was 23 years old. The youngest age was 19 years old. There were 172 female students and 63 male students, accounting for 73.2 and 26.8%, respectively. There were 13 freshmen students, accounting for 5.5%. There were 123 sophomores, accounting for 52.4%. There were 13 junior students, accounting for 5.5%. There were 86 seniors, accounting for 36.6%. There were 4 philosophy majoring students, taking up 1.7%; 4 economics majoring students, taking up 1.7%; 4 law majoring students, taking up 1.7%; 160 education majoring students, taking up 68.1%; 7 literature majoring students, taking up 3.0%; 32 science majoring students, taking up 13.6%; 14 engineering majoring students, taking up 6.0%; 2 medicine majoring student, taking up 0.8%; 5 management majoring students, taking up 2.2%; 2 arts majoring students, taking up 0.8%; 1 cross-disciplinary majoring student, taking up 0.4%.

The study was approved by the Ethics Committee of Qufu Normal University. Permission to conduct the study was applied and obtained from the administration of each selected school. Written informed consent was signed from the students of the selected schools prior to the survey. In the offline face-to-face approach, the researcher informed the students about the instructions on how to fill in the questionnaire and the precautions to be taken in filling in the questionnaire. The data would be used for research purposes only and would not affect their grades or studies. Participants were asked to provide truthful answers to each question, with no correct or incorrect answers. The online method used Questionnaire Star to collect the questionnaires, which also had detailed instructions and notes in the preface section of the questionnaire. Through a combination of online and offline methods, questionnaires were randomly distributed to college students of different grades and majors in colleges and universities using Questionnaire Star or paper-based questionnaires. For the convenience of statistical analysis, eventually, the paper version was collected and manually entered into Questionnaire Star. Questionnaire Star has the advantages of simple operation interface, offering various types of question options and robust collection channels, which is very suitable as a data collection tool. The results of the study will help to improve the quality of learners’ learning engagement.

### 3.2 Measurement

#### 3.2.1 Personality traits scale

This study was adapted from the NEO-FFI (NEO Five-Factor Inventory) developed by Costa and Mc Crae (1992). The NEO-FFI scale consists of a total of 60 question items categorized into 5 dimensions, namely, Neuroticism, Extraversion, Openness, Agreeableness, and Conscientiousness, with 12 question items per dimension. Question items with critical ratio CR values <3 or *p* > 0.05 were excluded by the extreme value method. Question items with a product-difference correlation coefficient of <0.4 or *p* > 0.05 with the total score were excluded by the question-item-to-total-score correlation method. Question items with weak relationships between common factors were removed by the factor analysis commonality method, when the commonality value was <0.2 or the factor loading was <0.4, the question items were removed. The specific process of elimination as well as the final results are shown in Table 1.

**Table 1:** Design of personality traits scale.

Big five personality traits	Question items	Reverse scoring question items	Remaining question items after extreme value method	Remaining question items after correlation of items with total score	Remaining question items after factor analyzing commonality method	Latest coding of question items
Neuroticism	1, 6, 11, 16, 21, 26, 31, 36, 41, 46, 51, 56	1, 16, 31, 46	6, 21, 36, 41, 51	6, 21, 51	6, 21, 51	6 → SJZ1, 21 → SJZ2, 51 → SJZ3
Extraversion	2, 7, 12, 17, 22, 27, 32, 37, 42, 47, 52, 57	12, 27, 42, 57	2, 7, 17, 37, 52, 57	2, 7, 17, 37, 52, 57	2, 17, 52, 57	2 → WQX1, 17 → WQX2, 52 → WQX3, 57 → WQX4
Openness	3, 8, 13, 18, 23, 28, 33, 38, 43, 48, 53, 58	18, 23, 28, 33, 48	8, 13, 28, 38, 43, 53	8, 13, 38, 53	8, 13, 38	8 → KFX1, 13 → KFX2, 38 → KFX3
Agreeableness	4, 9, 14, 19, 24, 29, 34, 39, 44, 49, 54, 59	9, 14, 19, 24, 39, 44, 54, 59	4, 14, 29, 34, 39	4, 14, 29, 34, 39	14, 29, 34, 39	14 → YRX1, 29 → YRX2, 34 → YRX3, 39 → YRX4
Conscientiousness	5, 10, 15, 20, 25, 30, 35, 40, 45, 50, 55, 60	15, 30, 45, 55	5, 10, 20, 25, 35, 40, 55, 60	5, 10, 20, 25, 35, 40, 55, 60	5, 10, 25, 35, 40, 55	5 → JZX1, 10 → JZX2, 25 → JZX3, 35 → JZX4, 40 → JZX5, 55 → JZX6

In this study, the personality traits scale had good reliability. First, the reliability was good (Ketchen, 2013). The Cronbach’s alpha coefficient of the Big Five personality scale was 0.904. The Cronbach’s alpha coefficients of Neuroticism, Extraversion, Openness, Agreeableness, and Conscientiousness were 0.911, 0.901, 0.905, 0.900, and 0.899, respectively. Second, the structural validity was good. The structural validity indicators were *Χ*^2^/df = 2.029, RMSEA = 0.061, GFI = 0.901, NFI = 0.911, IFI = 0.913, and CFI = 0.909. Third, the convergent validity met the requirements. Convergent validity refers to the degree of influence of a factor on other factors. Convergent validity values were determined by combining reliability (CR) and average extracted variance (AVE). The AVE values for Neuroticism, Extraversion, Openness, Agreeableness, and Conscientiousness in the Big Five Personality Scale in this study were 0.590, 0.574, 0.516, 0.521, and 0.510. The values of the combined reliability were 0.742, 0.773, 0.774, 0.743, and 0.805, which indicated that the convergent validity of each item met the desired goal. Fourth, the discriminant validity meets the standard. Discriminant validity means that there should be no strong correlation between different dimensions and that it is possible to distinguish different dimensions between scales. It is mainly measured by three kinds of data: correlation coefficients (r), AVE values, and arithmetic square root of AVE values among the latent variables. The correlation coefficients of the dimensions in this study are less than the arithmetic square root of the corresponding AVE, and the discriminant validity meets the standard. Therefore, the scale is reliable.

#### 3.2.2 Learning engagement scale

In this study, learning engagement was divided into three dimensions: behavioral engagement, affective engagement, and cognitive engagement based on the three-dimensional structure of learning engagement proposed by Fredricks and McColskey (2012). Behavioral engagement is the active participation behavior towards achieving positive academic activities. Affective engagement is students’ sense of belonging to school or positive and negative reactions to teachers and their peers. Cognitive engagement is the adoption of deep learning strategies during the learning process and willingness to work to understand complicated ideas and master difficult skills (Fredricks et al., 2004). The learning engagement survey scale draws on the findings of Ma et al. (2017), Gunuc and Kuzu (2014), Handelsman et al. (2005). The final version of the Learning Engagement Scale was also analyzed through item analysis, exploratory factor analysis, and reliability analysis.

The reliability of the scale is trustworthy. First, the reliability is good (Ketchen, 2013). The indicators were: behavioral engagement: Cronbach’s *α* = 0.928; emotional engagement: Cronbach’s α = 0.937; cognitive engagement: Cronbach’s α = 0.909; total scale: Cronbach’s α = 0.922. Second, the structural validity was good. The indicators were X2/df = 1.798, RMSEA = 0.071, GFI = 0.913, NFI = 0.919, IFI = 0.924, and CFI = 0.903. Third, the convergent validity met the requirements. The AVE values of behavioral engagement, affective engagement, and cognitive engagement in the learning engagement scale were 0.512, 0.503, and 0.548, and the combined reliability values were 0.777, 0.765, and 0.802. Fourth, discriminant validity met the criteria. The correlation coefficients of each dimension were less than the arithmetic square root of the corresponding AVE.

#### 3.2.3 Emotion regulation scale

The Classical Emotion Regulation Scale was used in this study. The Classical Emotion Regulation Scale was developed by Gross (2002) and later revised and translated into a Chinese version by Wang et al. (2007). The scale is divided into two strategies: cognitive reappraisal and expressive inhibition. Cognitive reappraisal is a strategy in which individuals regulate their emotions by re-understanding events. Expressive inhibition is the individual’s ability to control emotions through rationalization. The classical emotion regulation scale uses a seven-point Likert scale. Dawes (2008) found that comparing 5- point or 7-point data with 10-point data can be easily done with simple proportional and arithmetic adjustments. In this study, the scores on the 7-point scale were converted to scores on the 5-point scale for ease of data processing. Based on the formula used by Preston and Colman (2000): present scale score = (score-1)/(original scale-1) × scale to be transformed. In the present study, we needed to convert the 7-point scale to a 5-point scale, and this formula could be used by adapting the formula to the present scoring system score = (rating-1) / (7–1) × 5, thus reformatting all scales to a full score of 5. A feature of this approach is that any score using the lowest scale point on any scale will become a zero. For example, a score of 1 on a 7-point scale would become (1–1)/(7–1) × 5 = 0. Through this formula, all scores on a 7-point scale can be converted to scores on a 5-point scale. In this study, the Cronbach’s alpha coefficient for the emotion regulation scale was 0.896.

### 3.3 Data analysis

SPSS 27 and EXCEL 2016 were used for data processing and analysis in this study. Compared with other software, the above two software are much simpler to use, clearer in displaying results and more authoritative in analyzing data. Therefore, they were used as the data processing and analysis tools in this study. SPSS 27 software was used to analyze the collected data for reliability and validity, descriptive statistical analysis and difference statistical analysis. EXCEL 2016 software was used as an auxiliary tool for organizing all data. The mediating effect was carried out through Model 4 in the SPSS macro program process, and Bootstrap’s method was used to validate the mediating effect of analyzing emotion regulation strategies.

## 4 Results

### 4.1 Normal distribution test

In statistics, it is generally required that the data conforms to normal distribution, which is a prerequisite for carrying out data analysis. Common normal distribution test methods mainly include graphical methods, skewness coefficient and kurtosis coefficient checking method and K-S test and S-W test. In this study, skewness coefficient and kurtosis coefficient checking method were chosen to check the normal distribution of each question item of the data. A normal distribution is obeyed when the absolute value of skewness is less than 1.5 and the absolute value of kurtosis is less than 1.5 (Tabachnick and Fidell, 2019). According to Table 2, it can be seen that the skewness of all question items ranges from −1.474 to 1.195 and the kurtosis of all question items ranges from −1.274 to 1.499. Hence, all question items obeyed a normal distribution, which provided a good foundation for the subsequent analysis.

**Table 2:** Normal distribution test for each question item.

Question items	*M*	SD	skewness	kurtosis
SJZ1.I sometimes feel pain and indignant.	3.06	1.151	−0.193	−1.158
SJZ2. I often feel nervous and extremely upset.	2.66	1.080	0.516	−0.723
SJZ3. I often feel helpless and want others to solve my problems.	2.98	1.126	0.114	−1.047
WQX4. I like having lots of people with me.	3.06	1.102	−0.050	−0.986
WQX5. I enjoy talking to people.	3.23	1.060	−0.180	−0.919
WQX6. I am a very dynamic person.	3.16	1.092	−0.019	−1.132
WQX7. I prefer to act alone rather than lead others.	3.12	1.141	−0.218	−1.068
KFX8. I think it’s fun to take up new hobbies.	3.94	0.897	−1.002	0.811
KFX9. I am fascinated by art and natural expressive forms.	3.37	1.023	−0.200	−0.890
KFX10. I experience many different feelings and emotions.	4.07	0.731	−1.034	1.193
YRX11. Some people think I am selfish and self-centered.	3.61	1.000	−0.393	−0.501
YRX12. If I am insulted, I try to forgive and forget.	2.46	1.114	0.544	−0.582
YRX13. I tend to think the good side from people.	3.74	1.002	−0.897	0.439
YRX14. Some people think I am innocent and think only of myself.	3.57	1.028	−0.309	−0.590
JZX15. I pack and keep my things clean and tidy.	3.66	1.032	−0.659	−0.451
JZX16. I can organize my time well so that I get various things done on time.	3.40	1.000	−0.524	−0.580
JZX17. I have a clear set of goals and can achieve them gradually.	3.16	1.008	−0.203	−0.858
JZX18. I work hard in order to achieve my goals.	3.65	0.896	−0.719	0.148
JZX19. When I promise to do something, people always trust me to see it through.	3.59	0.889	−0.524	0.133
JZX20. I never seem to be able to keep things organized.	3.72	0.941	−0.789	0.099
XW1. I take the initiative to ask questions in class.	2.20	0.985	1.195	1.155
XW2. I listen carefully to other students in class.	3.73	0.893	−1.107	0.882
XW3. I will give fair comments on my classmates’ comments.	3.83	0.820	−1.226	1.499
XW4. I take good notes in class.	3.60	1.010	−0.980	0.378
XW5. I actively participate in group work.	3.82	0.835	−1.111	1.354
QG1. I am willing to share my learning feelings with my teacher and classmates.	3.20	1.147	−0.279	−1.098
QG2. I want to learn more through the course learning.	4.07	0.685	−1.381	1.479
QG3. I feel enjoyable and self of accomplishment in the learning process.	3.67	0.868	−0.845	0.651
QG4. I can well immerse into the atmosphere of classroom learning.	3.84	0.739	−1.142	1.198
QG5. I am enthusiastic about learning.	3.43	0.951	−0.474	−0.171
RZ1. I will adjust my study program at the right time according to my learning situation.	3.86	0.841	−1.474	1.466
RZ2. I will consider other possible alternative answers when I came up with one answer.	3.60	0.934	−0.783	−0.131
RZ3. I will make connections between what the teacher says and what I have learned previously to deepen my understanding.	3.66	0.903	−0.819	0.363
RZ4. I use different memory techniques (e.g., associative memory, repetition, etc.) to reinforce what I have learned.	3.80	0.856	−1.374	1.373
RZ5. I often reflect on my shortcomings in the learning process.	3.65	0.886	−0.955	0.535
1. When I want to feel some positive emotions (e.g., pleasant or happy), I will change the way I think about things.	3.80	0.794	−1.123	1.450
2. When I want to feel less negative emotions (e.g., sadness or anger), I will change the way I think about problems.	3.64	0.887	−0.814	0.238
3. When faced with a stressful situation, I will think about it in a way that is conducive to staying calm.	3.69	0.976	−0.781	−0.041
4. When I want to feel more positive emotions, I will change the way I think about the situation.	3.81	0.773	−0.840	0.980
5. When I want to feel less negative emotions, I will change the way I think about the situation.	3.69	0.838	−0.548	−0.187
6. I will control my emotions by changing the way I think about situations.	3.69	0.853	−0.897	0.864
7. When feeling positive emotions, I will be careful not to show them.	2.63	1.110	0.498	−0.883
8. I do not show my emotions.	2.54	1.099	0.492	−0.732
9. I control my emotions by not expressing them.	2.88	1.215	0.037	−1.274
10. When feeling negative emotions, I make sure not to reveal them to the outside world.	2.74	1.073	0.334	−0.869

### 4.2 Common method bias and multicollinearity diagnosis

In this study, Harman’s one-factor test was used to examine the collected data for common method bias (Podsakoff et al., 2003). Exploratory factor analysis was conducted on the unrotated factors to assess the results. Common method bias was present when the variance explained by the first common factor was greater than 40%. The results showed that there were 13 factors with eigenroots greater than 1. The explained variance of the first common factor was 16.012%. Consequently, this suggests that the data in this study do not exhibit significant common method bias. In addition, covariance diagnosis was performed to confirm the existence of multicollinearity among variables. The results showed that the variance inflation factor (VIF) values of all the question items were much less than the critical threshold of 3. Therefore, there is no problem of multicollinearity (Chennamaneni et al., 2016).

### 4.3 Descriptive statistics and correlation analysis of key variables

As shown in Table 3, the standard deviation of Big Five Personality scale is 0.399, the standard deviation of Learning Engagement scale is 0.485, and the standard deviation of Emotion Regulation scale is 0.494. Since 0.494 > 0.485 > 0.399, the data distribution of Big Five Personality scale is the most stable, while the data distribution of Learning Engagement scale is more stable than the data distribution of Emotion Regulation scale. Correlation analysis was conducted to find a significant correlation among Big Five Personality and learning engagement and emotion regulation. Significant correlations were found between emotion regulation and learning engagement as well. A correlation analysis of the dimensions of personality traits and emotion regulation with learning engagement reveals that Extraversion, Openness, Agreeableness, and Conscientiousness have significant positive correlations with learning engagement, while only Neuroticism has a negative correlation with learning engagement. Cognitive reappraisal and expressive inhibition were also significantly positively correlated with learning engagement. These results indicate that a mediated effects analysis is warranted, aligning with the predictions outlined in Hypothesis 1. Thus, Hypothesis 1 is supported.

**Table 3:** Descriptive statistics and correlation analysis of the main variables (*N* = 235).

	1	2	3	4	5	6	7	8	9	10	11	12	13
1. Neuroticism	1												
2. Extraversion	−0.064	1											
3. Openness	0.12	0.225**	1										
4. Agreeableness	−0.104	0.307**	0.119	1									
5. Conscientiousness	−0.151*	0.304**	0.257**	0.235**	1								
6. Behavioral engagement	−0.042	0.340**	0.265**	0.282**	0.389**	1							
7. Affective engagement	−0.091	0.301**	0.312**	0.222**	0.371**	0.602**	1						
8. Cognitive engagement	0.042	0.123	0.160*	0.108	0.314**	0.375**	0.495**	1					
9. Cognitive reappraisal	−0.185**	0.197**	0.216**	0.191**	0.188**	0.167*	0.339**	0.451**	1				
10. Expressive inhibition	−0.002	−0.146*	−0.109	−0.072	−0.085	0.078	0.125	0.138*	0.004	1			
11. Big five personality	0.200**	0.685**	0.525**	0.586**	0.700**	0.471**	0.422**	0.289**	0.230**	−0.150*	1		
12. Learning engagement	−0.037	0.309**	0.301**	0.247**	0.438**	0.790**	0.867**	0.779**	0.401**	0.142*	0.480**	1	
13. Emotion regulation	−0.135*	0.043	0.083	0.09	0.079	0.175**	0.331**	0.422**	0.729**	0.687**	0.065	0.388**	1
*M*	2.899	3.142	3.796	3.345	3.528	3.433	3.642	3.715	3.719	2.696	3.360	3.597	3.310
SD	0.816	0.789	0.618	0.707	0.641	0.536	0.633	0.620	0.598	0.845	0.399	0.485	0.494

*N* = 235, *p < 0.05, **p < 0.01, ***p < 0.001.

### 4.4 Differential analysis

Firstly, independent samples *t*-tests were conducted to analyze the difference among college students of different genders. Then one-way ANOVA was used to test for the difference analysis for college students of different grades and major subject disciplines. The results are shown in Table 4. There was no significant difference in the gender factor on the dimensions of neuroticism (*t* = −0.659, *p* = 0.511), extraversion (*t* = 0.809, *p* = 0.420), openness (*t* = −1.305, *p* = 0.193), agreeableness (*t* = 0.059, *p* = 0.953), conscientiousness (*t* = −0.410, *p* = 0.682), learning engagement (*t* = −1.150, *p* = 0.251), and emotion regulation (*t* = 0.352, *p* = 0.725). Similarly, the grade factor showed no significant difference in the grade level factor on the dimensions of neuroticism (*F* = 0.190, *p* = 0.903), extraversion (*F* = 0.586, *p* = 0.625), openness (*F* = 4.003, *p* = 0.080), agreeableness (*F* = 0.691, *p* = 0.558), conscientiousness (*F* = 1.195, *p* = 0.313), learning engagement (*F* = 1.305, *p* = 0.273), and emotion regulation (*F* = 1.056, *p* = 0.369). There was no significant difference in the academic discipline factor on the dimensions of neuroticism (*F* = 1.087, *p* = 0.373), extraversion (*F* = 0.755, *p* = 0.658), openness (*F* = 2.251, *p* = 0.200), agreeableness (*F* = 0.296, *p* = 0.975), conscientiousness (*F* = 0.855, *p* = 0.566), learning engagement (*F* = 0.542, *p* = 0.843) and emotion regulation (*F* = 0.979, *p* = 0.458). The results are shown in Table 4.

**Table 4:** Results of the test of variance.

	Classification	Number	Neuroticism	Extraversion	Openness	Agreeableness	Conscientiousness	Learning Engagement	Emotion Regulation
Genders	Male	63	2.84 ± 0.78	3.21 ± 0.78	3.71 ± 0.65	3.35 ± 0.68	3.5 ± 0.70	3.54 ± 0.57	3.33 ± 0.54
	Female	172	2.92 ± 0.83	3.12 ± 0.79	3.83 ± 0.60	3.34 ± 0.72	3.54 ± 0.62	3.62 ± 0.45	3.3 ± 0.48
	*t*		−0.659	0.809	−1.305	0.059	−0.410	−1.150	0.352
	*p*		0.511	0.420	0.193	0.953	0.682	0.251	0.725
Grade	Freshman	13	2.92 ± 0.75	3.23 ± 0.71	3.72 ± 0.51	3.27 ± 0.79	3.64 ± 0.42	3.7 ± 0.49	3.49 ± 0.46
	Sophomore	123	2.88 ± 0.83	3.13 ± 0.82	3.92 ± 0.58	3.29 ± 0.69	3.51 ± 0.64	3.54 ± 0.46	3.27 ± 0.51
	Junior	13	3.05 ± 0.85	2.88 ± 0.73	3.56 ± 0.53	3.38 ± 0.54	3.26 ± 0.6	3.63 ± 0.38	3.44 ± 0.46
	Senior	86	2.91 ± 0.81	3.18 ± 0.77	3.66 ± 0.66	3.43 ± 0.74	3.59 ± 0.67	3.66 ± 0.53	3.32 ± 0.48
	*F*		0.190	0.586	4.003	0.691	1.195	1.305	1.056
	*p*		0.903	0.625	0.080	0.558	0.313	0.273	0.369
Major subject discipline	Philosophy	4	2.83 ± 0.5	3.5 ± 0.5	3.75 ± 0.5	3.5 ± 0.5	3.08 ± 0.5	3.23 ± 0.5	3.45 ± 0.5
	Economics	4	3.08 ± 0.5	3.75 ± 0.5	3.67 ± 0.5	3.25 ± 0.5	3.46 ± 0.5	3.65 ± 0.5	3.45 ± 0.5
	Law	4	2.92 ± 0.5	3.13 ± 0.5	3.47 ± 0.5	3.44 ± 0.5	3.54 ± 0.5	3.23 ± 0.5	2.9 ± 0.5
	Education	161	2.86 ± 0.5	3.13 ± 0.5	3.89 ± 0.5	3.33 ± 0.5	3.56 ± 0.5	3.61 ± 0.5	3.3 ± 0.5
	Literature	7	3.62 ± 0.5	2.79 ± 0.5	3.95 ± 0.5	3.54 ± 0.57	3.57 ± 0.5	3.61 ± 0.5	3.17 ± 0.5
	History	0	0	0	0	0	0	0	0
	Science	32	2.98 ± 0.5	3.08 ± 0.5	3.48 ± 0.5	3.3 ± 0.5	3.39 ± 0.5	3.59 ± 0.5	3.3 ± 0.5
	Engineering	14	2.69 ± 0.5	3.18 ± 0.5	3.5 ± 0.5	3.45 ± 0.5	3.6 ± 0.5	3.61 ± 0.5	3.4 ± 0.5
	Agriculture	0	0	0	0	0	0	0	0
	Medicine	2	2.33 ± 0.5	3 ± 0.5	3.67 ± 0.5	2.88 ± 0.5	2.83 ± 0.5	3.6 ± 0.5	4 ± 0.5
	Military science	0	0	0	0	0	0	0	0
	Management	5	3.13 ± 0.5	3.6 ± 0.52	3.93 ± 0.5	3.55 ± 0.5	3.83 ± 0.5	3.71 ± 0.5	3.44 ± 0.5
	Arts	2	3.5 ± 0.5	2.88 ± 0.5	3.33 ± 0.5	3.5 ± 0.5	3.42 ± 0.5	3.57 ± 0.5	3.2 ± 0.5
	Interdisciplinary	0	0	0	0	0	0	0	0
	*F*		1.087	0.755	2.251	0.296	0.855	0.542	0.979
	*p*		0.373	0.658	0.200	0.975	0.566	0.843	0.458

### 4.5 The mediating role of emotion regulation strategies in the relationship between Big Five Personality Traits and learning engagement

**Figure 2:**
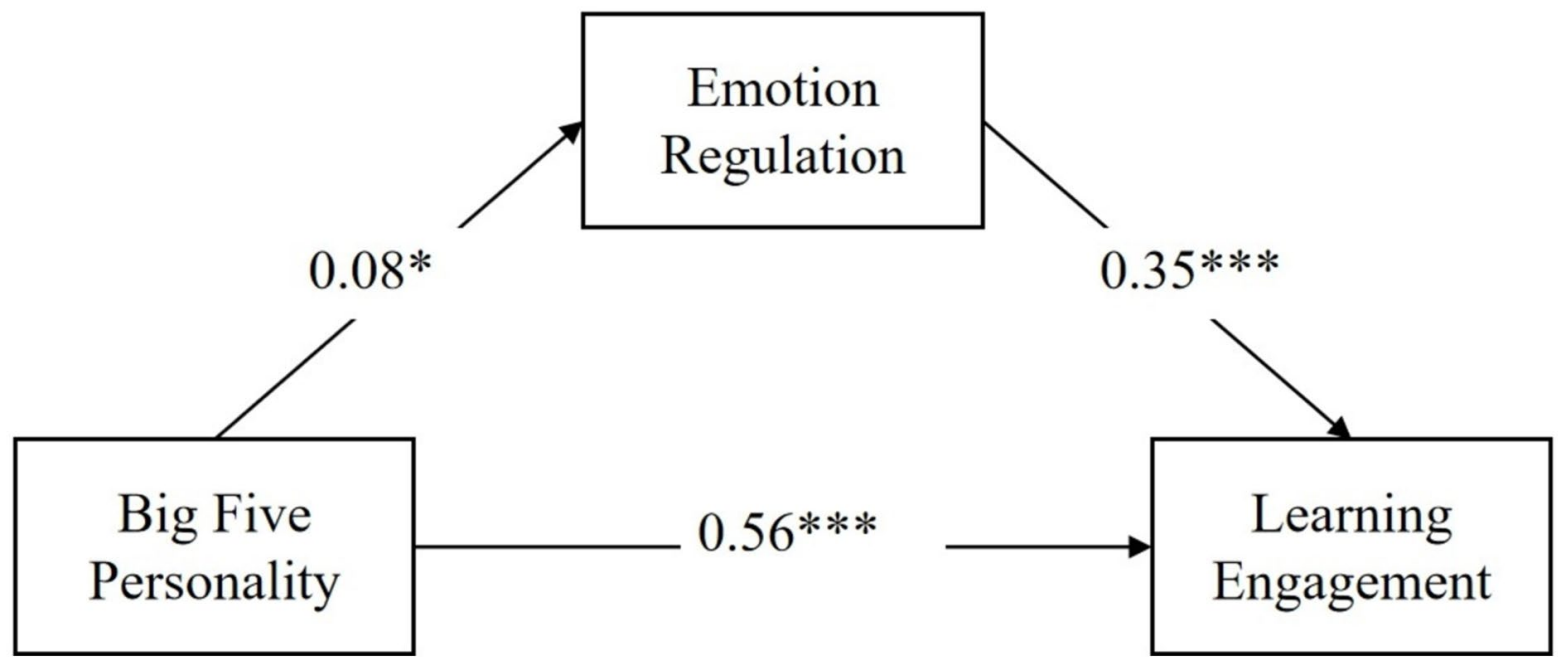
Path coefficient plots of Big Five Personality, emotion regulation strategies and learning engagement.

To explore the underlying mechanism of the influential role of Big Five Personality Traits on learning engagement, emotion regulation strategy was introduced as a mediating variable to substitute into the model in the study. The test of mediating effect was conducted by using Model 4 in the SPSS macro program process. The Bootstrap method provided by Hayes was used to validate the analysis of the mediating role of emotion regulation strategies between Big Five personality traits and learning engagement. The path coefficients of emotion regulation strategies between the Big Five Personality Traits and learning engagement variables are shown in Figure 2.

According to Table 5, the Bootstrap 95% confidence interval’s lower and upper limits for the mediating effects of emotion regulation strategies on Big Five personality traits and learning engagement do not contain 0, indicating that Big Five personality traits have a direct effect on learning engagement and also exert a mediating effect through emotion regulation strategies. This direct effect (0.556) and mediating effect (0.028) accounted for 95.21 and 4.79% of the total effect (0.584). This result is consistent with the prediction of hypothesis 2. Thus, hypothesis 2 is confirmed.

**Table 5:** Table of total, direct and mediating effects.

	Efficacy value	SE	LLCI	ULCI	Percentage
Total effect	0.584	0.070	0.447	0.722	
Direct effect	0.556	0.064	0.430	0.683	95.21%
Mediating effect	0.028	0.041	0.055	0.105	4.79%

*p < 0.05, **p < 0.01, ***p < 0.001.

## 5 Discussion

This study investigates college students to investigate the effects of personality traits on learning engagement and the mediating role of emotion regulation strategies on this effect.

### 5.1 Current status of college students’ personality traits, learning engagement and emotion regulation strategies

In terms of exploring the personality traits of college students in depth, this study found through exhaustive data analysis that college students have a relatively high mean score (*M* = 3.796) on the personality trait of Openness. This is a good indication that they possess an unusually active imagination, not only are they willing to accept all kinds of new ideas and new things, but also show endless curiosity and desire to explore the unknown world. On the dimension of Conscientiousness, the mean score (*M* = 3.528) of college students is also high. This reflects that they have excellent self-control in daily life, can effectively manage and regulate their impulsive behaviors, are more cautious and considerate in doing things, and show a high sense of responsibility and maturity (Steiner et al., 2023). The mean score (*M* = 3.345) of college students was also more prominent on the trait of Agreeableness. This means that they show more understanding and generosity in the process of getting along with their classmates and friends, are always helpful and good at building harmonious interpersonal relationships. The mean score of Extraversion (*M* = 3.142) was found to be at a moderate level in this study. This result reveals a relatively balanced distribution of Extraversion and Introversion personalities among college students. They are able to present themselves positively in social situations as well as maintain inner peace and solitude when needed. In contrast, the mean score for the dimension of Neuroticism (*M* = 2.899) was the lowest. This further indicates that college students are generally calm. They were able to remain calm and steady in the face of various situations, were not easily overly influenced by emotions, and demonstrated good psychological adjustment ability. In terms of learning engagement, this study also draws encouraging conclusions. College students had higher mean scores on the dimensions of cognitive engagement (*M* = 3.715), emotional engagement (*M* = 3.642), and behavioral engagement (*M* = 3.433). This is a good proof that they are more adept at using cognitive and metacognitive strategies to dig deeper into the deeper meaning of knowledge for meaningful learning constructs. During the learning engagement, they are able to give their full attention to the learning activities, put in unremitting efforts for them, and always maintain a positive emotional response. This state of engagement will undoubtedly help them achieve better academic performance (Lang et al., 2022). In addition, in terms of the use of emotion regulation strategies, this study also found that college students had relatively high mean scores (*M* = 3.719) on the dimension of cognitive reappraisal, which suggests that they are more inclined to regulate their emotional state by re-cognizing the event, demonstrating a rational and mature way of coping. In contrast, the mean score on the dimension of expression inhibition (*M* = 2.696) is at a moderate level, which indicates that college students are more inclined to choose to reconstruct things positively when regulating their emotions rather than simply inhibit their emotional expression, a tendency that undoubtedly reflects their more positive and healthy psychological state (Zhao et al., 2016).

This study found that there was no significant difference in Big Five personality, learning engagement, and emotion regulation across gender, grade level, and subject discipline. This is inconsistent with the findings of previous studies. For example, Xu et al. (2023) discovered that there was a significant difference in learning engagement between grades. Yang (2020) found that male students used expressive inhibition strategies more frequently than female students. In her study, Zhang (2022) found that the level of learning engagement differed between genders. This inconsistency in findings may be a result of differences in research participants and research settings. Meanwhile, college students have matured mentally, have passed the impulsive period of adolescence, are emotionally desensitized, and are able to make a full commitment to learning engagement. Regardless of grade level or major, they are able to perceive the implementation of learning and to regulate their emotions during the learning process.

### 5.2 The relationship between college students’ personality traits and learning engagement

Research indicates that both Extraversion and Agreeableness have a significant positive correlation between behavioral engagement and emotional engagement. This means that students who are extroverted and lively, sociable as well as helpful and empathetic tend to show more positive behavioral engagement and rich emotional engagement in the learning process (Mahama et al., 2022; Zhao and Ji, 2024). Meanwhile, both traits of Openness and Conscientiousness showed significant positive correlations with behavioral engagement, emotional engagement, and cognitive engagement. This shows that students with open-mindedness, openness to new things as well as rigor and conscientiousness, and dutifulness are able to engage in learning engagement in all aspects, not only showing a high degree of concentration in action and emotion, but also thinking deeply and exploring actively at the cognitive level (Sahinidis et al., 2020). It is worth noting that there is a negative correlation between only one trait, Neuroticism, and learning engagement. Such results have been obtained by other researchers (Laidra et al., 2007; Barrick et al., 1998). Consequently, students who excel at teamwork, and face challenges with optimism and positivity, while approaching knowledge with modesty and caution, tend to be more effective learners. They are able to think well and willing to make unremitting efforts to find solutions when facing difficulties and challenges in learning, and show strong stress resistance. They have higher learning engagement in the learning process (Morfaki and Skotis, 2022; Quigley et al., 2022; Zhang et al., 2020).

### 5.3 The relationship between emotion regulation and learning engagement

Research indicates that there is a significant positive correlation between each emotion regulation strategy in terms of students’ learning engagement, affective engagement, and cognitive engagement (Santos et al., 2021). Effective utilization of these emotion regulation strategies can greatly enhance students’ overall learning engagement in the learning process. When individuals inevitably encounter various problems and challenges in their studies, work, and life, they are often prone to negative emotions such as anxiety, restlessness, and irritability (Pan, 2023). If left unregulated, these negative emotions can significantly impede students’ learning engagement. Notably, there are substantial differences in emotion regulation abilities among individuals (Shafiee Rad and Jafarpour, 2022). Individuals with stronger emotion regulation abilities are usually more adept at flexibly utilizing a variety of effective regulation strategies to quickly adjust their state of mind to a normal state, and then are able to engage in learning engagement with more enthusiasm and higher efficiency (Yu et al., 2022); in contrast, individuals with weaker emotion regulation abilities tend to have difficulties in adjusting from negative emotions, which can adversely affect their learning outcomes (Bielak and Mystkowska-Wiertelak, 2020). Therefore, individuals with better emotion regulation tend to be better able to regulate their emotions and thus effectively improve their learning engagement.

### 5.4 The mediating role of emotion regulation

Research indicates that emotion regulation plays a significant partial mediating role between personality traits and learning engagement in college students. This further reveals the complex mechanism of the influence of personality traits on learning engagement. This finding is consistent with previous studies (Huang et al., 2023; Zhang et al., 2024). Specifically, personality traits not only directly affect learning engagement, but also influence it indirectly via emotion regulation. Individuals with high Openness, Conscientiousness, and Agreeableness are able to show a spirit of courage to face challenges and defy difficulties when facing heavy learning tasks or sudden learning difficulties. They are less likely to fall into pessimistic and negative moods. These individuals usually have strong self-confidence and can experience a sense of achievement and satisfaction in the learning process, thus gaining more positive emotions. These positive emotions, in turn, motivate them to focus more on their studies, forming a virtuous cycle.

In stark contrast, individuals low in Openness, Conscientiousness, and Agreeableness are more susceptible to negative emotions such as frustration and anxiety when confronted with academic tasks. These emotions bind them, making it difficult for them to enter a normal learning state, and may even lead to a serious setback in learning motivation (Brady et al., 2018). It is worth noting that even under negative emotions, if individuals can effectively master and flexibly use emotion regulation strategies, they still have the opportunity to adjust their own emotional state, gradually get rid of the plague of negative emotions, and then enhance learning engagement, injecting new vitality and hope into the learning process (Gong, 2023). This process not only reflects the importance of emotion regulation, but also emphasizes the far-reaching significance of cultivating good personality traits and enhancing emotion regulation for improving learning engagement.

## 6 Contribution and implications

This study has important implications for the enhancing of college students’ learning engagement. This study shows that there is a strong relationship between college students’ personality traits, emotion regulation strategies and learning engagement. The personality traits of college students directly influence learning engagement. Emotion regulation partially mediates the relationship between personality traits and learning engagement. Students who excel at teamwork, optimistic and positive, modest and prudent, and responsible and self-disciplined are more engaged in the learning process, and are better at adopting emotion regulation strategies to regulate their emotions in the face of learning challenges, which is more conducive to learning activities. Therefore, in the process of education, teachers not only need to teach students according to their different personality traits, but also cultivate and shape students’ positive personality traits, and actively guide students to use emotion regulation strategies to eliminate negative emotions, so that students can be happily engaged in learning activities. School counselors can also use psychological counseling skills to provide personalized psychological counseling services for college students, guiding college students to meet difficulties and challenges with an optimistic attitude.

## 7 Limitations and future research

This study has certain limitations regarding the research tools employed. This is manifested in the fact that it only relies on a single research tool, the questionnaire, to collect and analyze data, and does not make full use of a variety of other research methods, such as the interview method and the experimental method, as auxiliary means to enrich the perspective and depth of the study. This single way of using research tools may limit the comprehensiveness and accuracy of the research results to a certain extent. In order to compensate for this deficiency, in our future research, we will be committed to comprehensively utilizing a variety of investigative tools and methods, including, but not limited to, in-depth interviews, laboratory experiments, case studies, etc., with a view to exploring in depth the relationship between personality traits, learning engagement, and emotion regulation from a variety of dimensions and levels so as to further enhance the reliability and validity of the research results. Additionally, there are limitations related to the sample selection. The sample of college students we selected was mainly concentrated in colleges and universities in Shandong Province, which makes the geographic distribution of the data relatively narrow and may not fully reflect the real situation and differences in personality traits, learning engagement and emotion regulation among college students nationwide. In order to overcome this limitation, future research should further expand the coverage of the sample data and extend the study object to students in colleges and universities in other regions or even nationwide, so as to more comprehensively analyze and explore the relationships among personality traits, learning engagement and emotion regulation among college students in different regions, as well as the differences and commonalities of these relationships in the context of different geographic and cultural backgrounds. Conducting cross-regional and cross-cultural comparative studies could yield more comprehensive, in-depth, and generalized conclusions, providing a stronger scientific basis and reference for educational practice and policy development in related fields.

## 8 Conclusion

This study sheds light on the impact of college students’ personality traits on learning engagement and highlights the mediating role of emotion regulation. The results of the study showed a significant correlation between college students’ personality traits and learning engagement. Students who are cooperative, optimistic and positive, modest and prudent, and responsible and self-disciplined are more likely to be engaged in learning activities. Emotion regulation mediates the relationship between personality traits and learning engagement. Appropriate use of emotion regulation strategies can improve mindfulness and help learning engagement. Thus, the results of this study hold considerable significance for enhancing college students’ learning engagement.

## Data Availability

The original contributions presented in the study are included in the article/supplementary material, further inquiries can be directed to the corresponding author/s.
